# Application of simultaneous selective pressures slows adaptation

**DOI:** 10.1111/eva.13062

**Published:** 2020-08-15

**Authors:** Lauren M. F. Merlo, Kathleen Sprouffske, Taylor C. Howard, Kristin L. Gardiner, Aleah F. Caulin, Steven M. Blum, Perry Evans, Antonio Bedalov, Paul D. Sniegowski, Carlo C. Maley

**Affiliations:** ^1^ Lankenau Institute for Medical Research Wynnewood Pennsylvania USA; ^2^ Disease Area Oncology Novartis Institutes for BioMedical Research Basel Switzerland; ^3^ Department of Pathology and Laboratory Medicine UC Davis Health Sacramento California USA; ^4^ School of Veterinary Medicine University of Pennsylvania Philadelphia Pennsylvania USA; ^5^ PEEL Therapeutics, Inc. Salt Lake City Utah USA; ^6^ Department of Medical Oncology Dana‐Farber Cancer Institute Broad Institute at MIT and Harvard Harvard Medical School, and Massachusetts General Hospital Cancer Center Boston Massachusetts USA; ^7^ Department of Biomedical and Health Informatics Children's Hospital of Philadelphia Philadelphia Pennsylvania USA; ^8^ Clinical Research Division Fred Hutchinson Cancer Research Center Seattle Washington USA; ^9^ Department of Biology University of Pennsylvania Philadelphia Pennsylvania USA; ^10^ Arizona State University School of Life Sciences Biodesign Institute Tempe Arizona USA

**Keywords:** adaptation, cancer therapy, clonal interference, functional interference, slowing evolution

## Abstract

Beneficial mutations that arise in an evolving asexual population may compete or interact in ways that alter the overall rate of adaptation through mechanisms such as clonal or functional interference. The application of multiple selective pressures simultaneously may allow for a greater number of adaptive mutations, increasing the opportunities for competition between selectively advantageous alterations, and thereby reducing the rate of adaptation. We evolved a strain of *Saccharomyces cerevisiae* that could not produce its own histidine or uracil for ~500 generations under one or three selective pressures: limitation of the concentration of glucose, histidine, and/or uracil in the media. The rate of adaptation was obtained by measuring evolved relative fitness using competition assays. Populations evolved under a single selective pressure showed a statistically significant increase in fitness on those pressures relative to the ancestral strain, but the populations evolved on all three pressures did not show a statistically significant increase in fitness over the ancestral strain on any single pressure. Simultaneously limiting three essential nutrients for a population of *S. cerevisiae* effectively slows the rate of evolution on any one of the three selective pressures applied, relative to the single selective pressure cases. We identify possible mechanisms for fitness changes seen between populations evolved on one or three limiting nutrient pressures by high‐throughput sequencing. Adding multiple selective pressures to evolving disease like cancer and infectious diseases could reduce the rate of adaptation and thereby may slow disease progression, prolong drug efficacy and prevent deaths.

## 1 INTRODUCTION

Methods for slowing the rate of adaptation of cell populations would be valuable tools for preventing human suffering from evolving diseases including both infectious diseases and cancer. The evolution of drug resistance is a major problem worldwide for cancer therapy, antibacterial agents, antifungal agents, antimalarial agents, as well as pesticides and herbicides (Barzman et al., 2015; Hughes & Andersson, 2015; Tabashnik, 1989). Methods for slowing the evolution of drug resistance would thus save lives as well as produce significant economic benefits in agriculture, through improved pest and weed management. There are several known evolutionary mechanisms that may affect rates of adaptation. These include variations in mutation rate (Arjan et al. 1999; Sprouffske, Aguilar‐Rodríguez, Sniegowski, & Wagner, 2018), as well as the other parameters of the rate of evolution such as population size and generation time (Maley, Szabo, & Reid, 2011), Hill‐Robertson interference affecting evolution at linked sites (Hill & Robertson, 1966; McVean & Charlesworth, 2000), fluctuating selection (Cvijović et al., 2015), epistasis (Chou, Chiu, Delaney, Segrè, & Marx, 2011; Perfeito, Sousa, Bataillon, & Gordo, 2014), and genetic and functional interference, which predict that competition between different clones may alter the rate of adaptation. Functional interference is essentially antagonistic pleiotropy. This occurs when mutations that confer an advantage in one environment have a fitness cost in another environment (Bell 2008). Antagonistic pleiotropy is often invoked as reason for the limits of selection and adaptation (Williams 1957; Orr 2000; Hong and Nielsen, 2013) particularly in explaining the evolution of specialists vs. generalists (Elena, Agudelo‐Romero, & Lalić, 2009; Nikolin et al. 2012). Clonal interference theory predicts that multiple beneficial mutations may arise and compete within a population, effectively slowing the time to fixation of adaptive mutations in asexual populations (Desai et al., 2007; de Visser & Rozen, 2006) such as neoplastic cells. Although a prediction of interference theory, this has rarely been directly investigated. Here, we aim to directly test one possible mechanism for reducing the rate of adaptation: evolution under multiple, competing selective pressures.

Multiple competing selective pressures are undoubtedly of both ecological relevance, as organisms are likely facing multiple pressures at any given time, as well as applicable to understanding both progression and therapy in human disease, including cancer and infectious diseases (Strelkowa & Lässig, 2012). In particular, a cancer accumulates large numbers of mutations and progresses through a complex evolutionary process of clonal selection and expansion (Attolini & Michor, 2009; Greaves & Maley, 2012; Maley et al., 2017; Merlo et al., 2006), meeting many of the conditions required for clonal and functional interference (Illingworth & Mustonen, 2011; Sprouffske et al., 2012). If we can effectively alter the rate of evolution through the application of multiple selective pressures, we can slow tumor progression and the evolution of therapeutic resistance. Existing empirical studies of clonal interference are of replicate populations in a single environment (de Visser & Rozen, 2006; Gerrish & Lenski, 1998; Kao & Sherlock, 2008; Rozen et al., 2002) and do not directly address the role of simultaneous selective pressures on the rate of evolution. Additionally, evolution in complex environments has infrequently been directly examined experimentally (Barrett et al., 2005; Lunzer et al., 2002).

We have chosen to study this phenomenon in a *S. cerevisiae* model system, a common system for the development of anti‐cancer agents (Pereira et al., 2012; Simon & Bedalov, 2004). Here, our selective pressures are various nutrient limitations: glucose, a carbon source; histidine, an essential amino acid; and uracil, a nucleotide. These nutrients were chosen because the biosynthesis pathways of histidine and uracil have little overlap and are generally separate from the central metabolic pathways of glucose metabolism. We have examined the relative rate of adaptation of *S. cerevisiae* to limiting glucose, histidine, uracil, and the combination of all three during a ~500 generation batch culture evolution experiment. We measure the rate of adaptation by quantifying the increase in fitness over that time period. We sought to reduce the likelihood of "generalist" mutations that confer an advantage under all three conditions by choosing pressures with no known overlap in the biosynthetic pathways of these nutrients. Rather, “specialist” mutations conferring an advantage to each nutrient limitation should be more likely in this experiment. In accordance with the theoretical predictions of reduced rates of adaptation under multiple selective pressures, we observed that populations evolved under multiple pressures were less adapted to growth under any single pressure than the populations evolved under only a single selective pressure.

When a population evolved under a single selective condition, it evolved a higher fitness on that condition than the ancestral strain. This is in keeping with well‐established principles of natural selection and has been amply demonstrated by experimental evolution. However, populations evolved under multiple pressures did not statistically significantly improve in fitness relative to the ancestral strain for any of the given pressures. To show that fitness improvements were not due to general adaptation to batch culture conditions, we verified that evolved populations had the highest fitness when grown on the media used for their evolution and in most cases did not show consistent fitness gains in other media. This work provides a direct empirical demonstration that evolution under multiple selective pressures slows the rate of adaptation, a phenomenon critical to our understanding of disease evolution, resistance, and development of novel therapies.

## 2 METHODOLOGY

### 2.1 Long‐term evolution

A single colony of an S288c haploid strain of *Saccharomyces cerevisiae* (MATα his3Δ1 leu2Δ0 lys2Δ0 ura3Δ0) was picked from a plate of YPD medium (20 g/L glucose, 20 g/L peptone, 10 g/L yeast extract, 15 g/L Bacto agar) and grown overnight in SD media (6.7 g/L Bacto Yeast Nitrogen Base, 20 g/L D‐glucose) supplemented with 20 mg/L L‐histidine, 100 mg/L L‐leucine, 30 mg/L L‐lysine, and 20 mg/L uracil. Following this, a small inoculum was transferred to 10 ml fresh media with limiting glucose (47.5 mg/L), limiting histidine (0.38 mg/L), limiting uracil (0.325 mg/L), or a combination of all three limitations at the concentrations above. These concentrations of nutrients were chosen after careful study to determine conditions that limited the growth of the cells to an approximately equal low growth rate and carrying capacity. Each day, 10 ml cultures were diluted 1/8 such that cultures underwent three doublings/day. Frozen stocks were generated at regular intervals by freezing in 25%–33% glycerol at −80°C. We started a second set of limiting cultures (replicate B) using frozen stocks from the first day's growth of replicate A on limiting media, such that the first three generations of evolution occurred in common before the replicates were generated. We would have liked to run more replicates, but due to the labor‐intensive and long‐term nature of the experiment, requiring daily management without break for 5 months, that was not feasible. We evolved the yeast for a total of 552 generations for replicate A and 507 generations for replicate B.

### 2.2 Measuring fitness

Batch culture competitions were performed to determine fitness because the carrying capacity of the nutrient‐limited cultures is too low to allow for reliable OD readings and calculation of growth rates for fitness measurements. Evolved populations and a G418‐sulfate‐resistant reference strain (MATα his3::kanmx leu2Δ0 lys2Δ0 ura3Δ0) were grown overnight in SD media supplemented with Ura, His, Leu, and Lys as described above. The reference strain was used because there are no differential selectable markers on the evolved populations to allow differentiation between populations in direct competition. Approximately equal numbers of the evolved population and reference strain were mixed, washed, and transferred to fresh media with limiting nutrients. Aliquots of this initial mixture were plated onto YPD plates and incubated 48 hours at 30°C. Batch culture competitions were then incubated for 24 hours at 30°C, 220rpm in a shaking incubator. After 24 hours, the overnight cultures were diluted and plated onto YPD as described. Three plates containing between ~ 15 and 150 colonies each from both 0 and 24‐hour time points were replica‐plated onto YPD/G418 sulfate and again onto YPD as a transfer control. Plates were incubated overnight and the proportion of evolved population and reference strain colonies was calculated from the total number of reference strain colonies growing on the three G418 sulfate replica plates. Relative fitness (W_evol/ref_) was calculated by taking the ratio of the Malthusian parameters (m) of the evolved population (m_evol_) and reference strains (m_ref_) (Lenski et al., 1991):

$$W_{\text{evol}/\text{ref}}=m_{\text{evol}}/m_{\text{ref}}$$

$$m=\ln(N_{t}/N_{0})/t$$

where N_0_ = initial population size and N_t_ = population size at t = 1 day (24 hours) as determined from colony counts based on dilution series.

### 2.3 Statistical analysis

Statistical comparisons were performed using R statistics software. We took a conservative approach and used nonparametric Wilcoxon rank sum tests to compare the fitness values with a simple Bonferroni correction for multiple testing, α < 0.05/29 = 0.0017. Initial tests determined whether A and B replicates were statistically significantly different. Subsequent tests combined replicates where there was no significant difference between the replicates.

### 2.4 High‐throughput sequencing

Evolved yeast populations and the ancestral strain were grown overnight in YPD media and genomic DNA prepped with Qiagen PureGene Yeast/Bact. Kit A. Genomic DNA was fragmented by sonication in a Biorupter (Diagenode) device. Genomic DNA libraries were prepared by the Wistar Institute Genomics Facility according to standard Illumina library preparation protocols and run on the Illumina GAII according to standard practices. The nine yeast populations (eight evolved and one ancestral) were multiplexed onto three lanes of a flow cell and 36‐base pair, paired end reads collected.

Illumina GAII reads were analyzed for point mutations and insertions/deletions per methods previously described (Araya et al., 2010). Briefly, an average of 7.49 × 10^6^ reads were collected from each of the 9 populations. Each read was aligned individually with Bowtie 0.12.2 (Langmead et al., 2009) to the 2008 *S. cerevisiae* genome (downloaded from the UCSC Genome Browser). Only uniquely aligning reads were considered in the downstream analyses. SAMtools software was used to remove PCR duplications and call point mutations present in evolved populations but not the ancestral strain. Mutations were only called if they were at fixation in the evolved population (i.e., there were no ambiguous base calls) and at ≥5x coverage at the site in both the ancestral strain and evolved population being compared. Duplications and deletions were identified by comparing the depth of coverage at each base in the ancestor and evolved populations. The raw coverage values were normalized by the total number of reads uniquely mapping to the genome for each population. For each base position, we calculated the ratio of the normalized coverage in the evolved population to the normalized coverage in the ancestral WT strain. To reduce the size of the computational problem so that a segmentation algorithm could run efficiently, we took the median of windows of size 11. To call breakpoints for regions of gain and loss in the evolved populations, the log_2_(median) values were used as input into the R package *DNAcopy* (version 1.24.0) which implements the Circular Binary Segmentation algorithm which has been used for similar data (Langmead et al., 2009). The data were smoothed by removing segments less than three standard deviations apart, and only breakpoints containing more than 1,000bps were considered for further analysis. On average, 93.35% of the genome was covered at an average depth of 24.4 reads per base (see Table S1 for sequencing results for individual populations).

## 3 RESULTS

### 3.1 Fitness results

**Figure 1:**
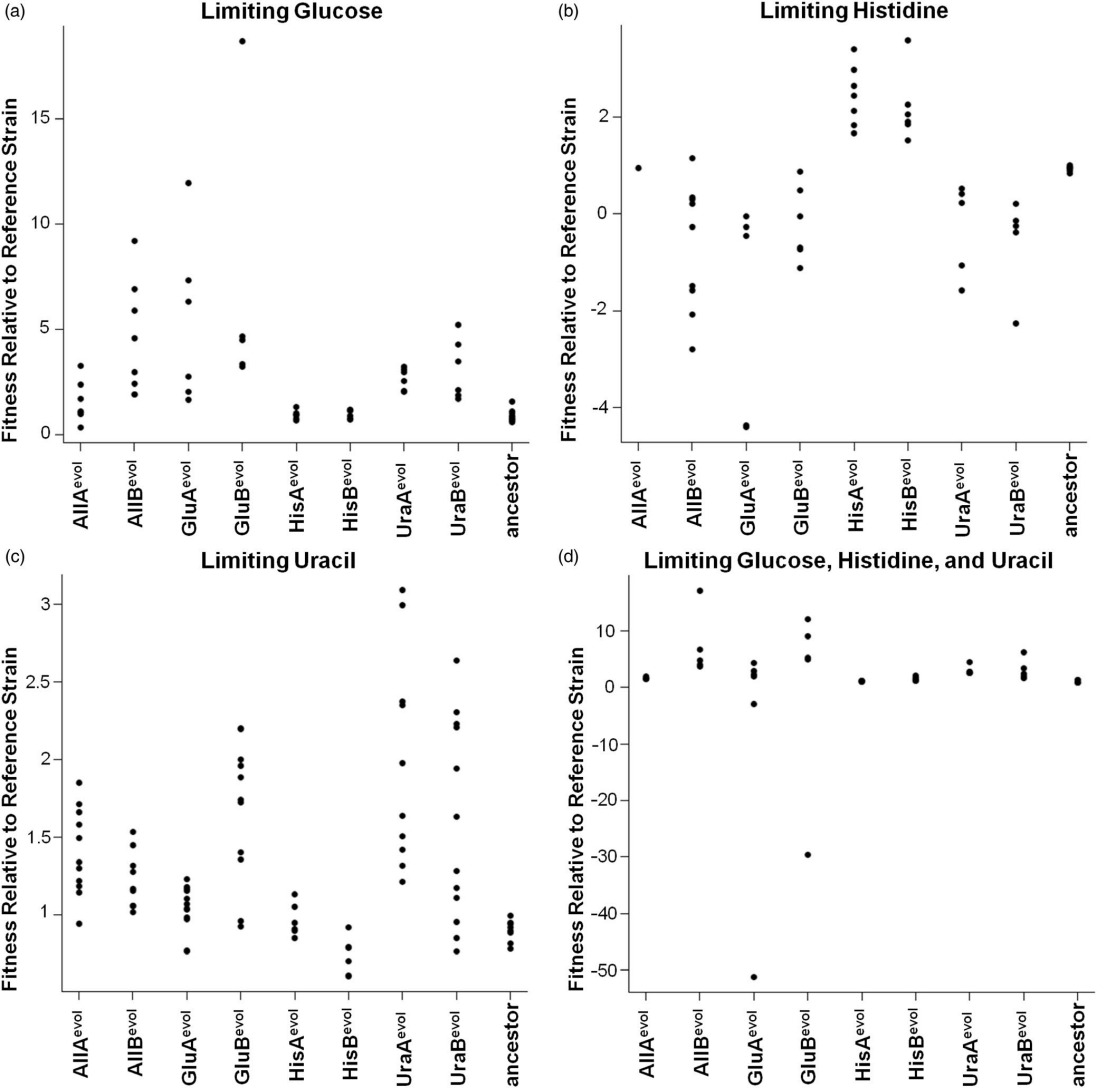
Relative fitness of eight evolved populations and ancestral strain on single limiting nutrients as well as the combination of three limiting nutrients. Each dot presents an independent fitness measurement from each evolved population. Note the difference in scale (y‐axis) in each of the four graphs

**Figure 2:**
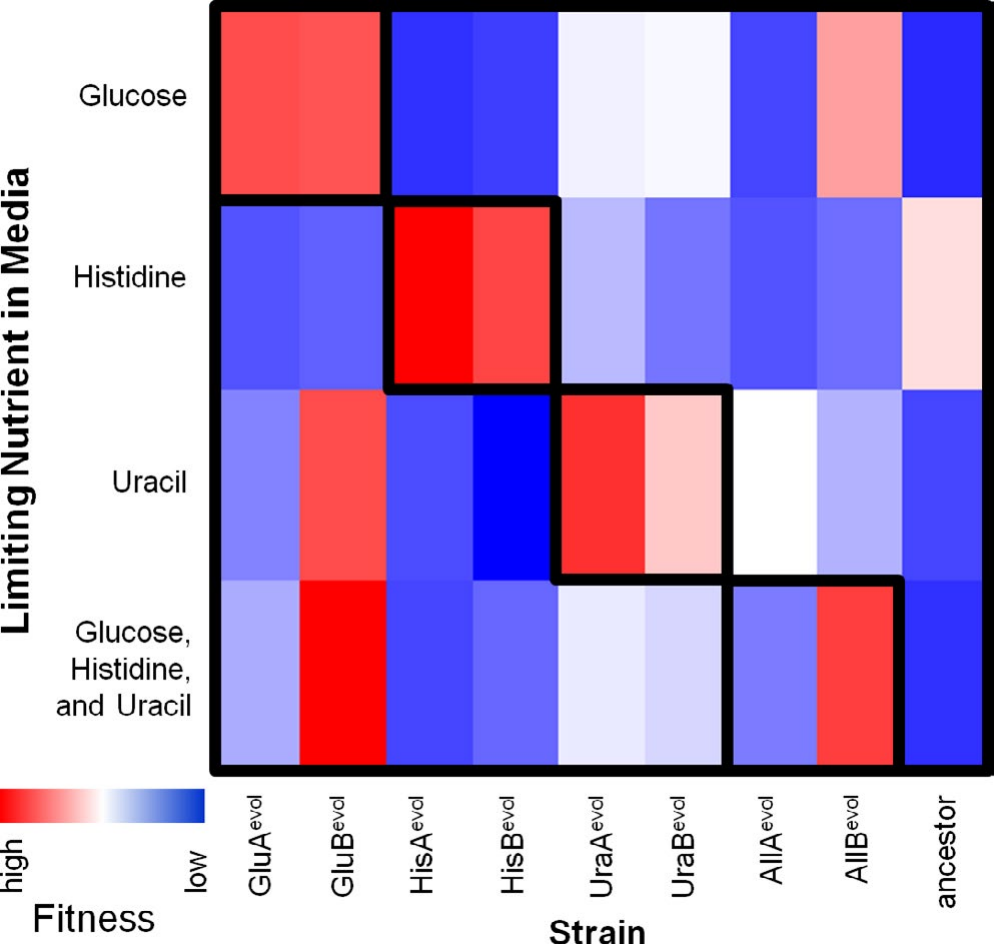
Heat maps of relative fitness of each population on the four experimental limiting media conditions. The relative fitness values were normalized for each experimental limiting media condition to aid visual interpretation. Highest fitness on each limitation = red, lowest = blue. Boxes indicate fitness on media under which each set of populations was evolved

**Figure 3:**
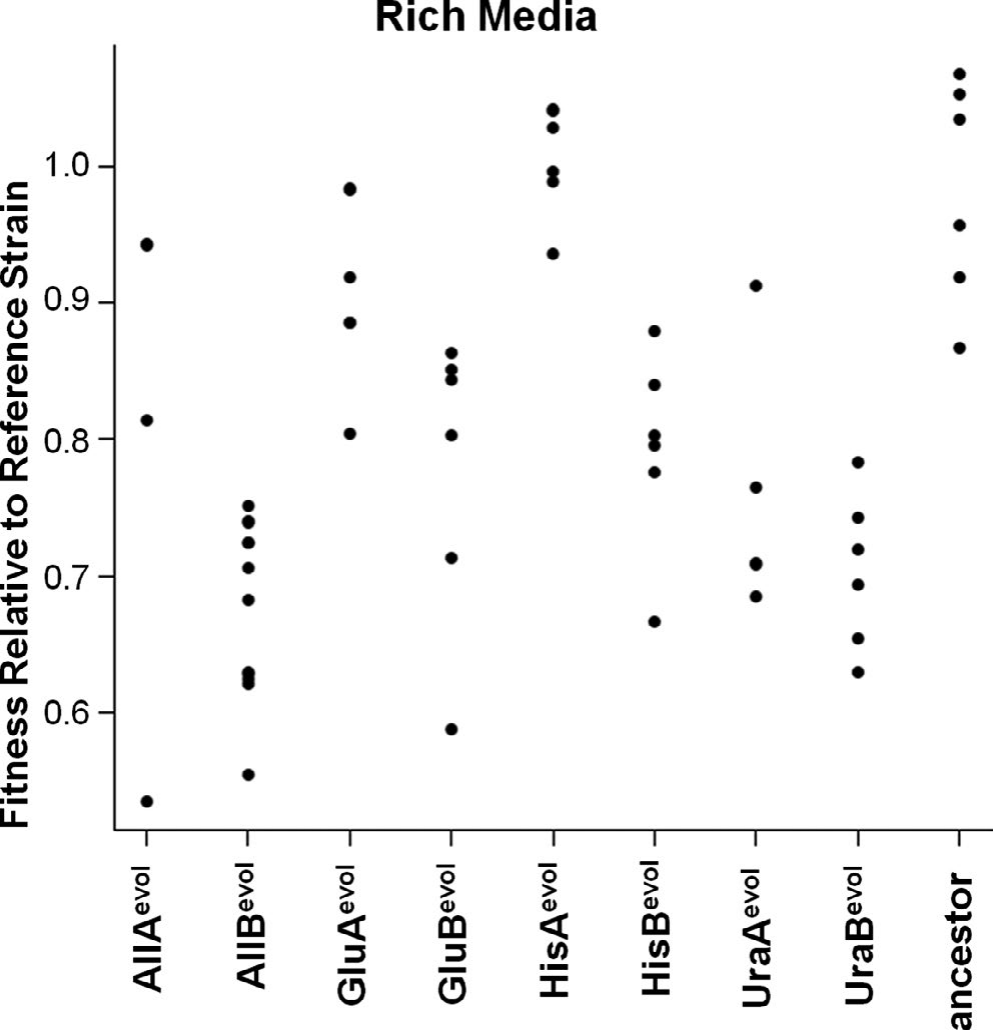
Relative fitness of evolved populations relative to reference strain on rich (YPD) media. Note that the relative fitness of the ancestor is centered around 1, with most evolved populations showing lower fitness on rich media than the ancestral strain, suggesting an evolutionary trade‐off between adaptations to limiting conditions vs. rich media

Fitness of all eight evolved populations and the ancestral strain was evaluated on a medium containing either limiting glucose, limiting histidine, or limiting uracil media as well as a medium limiting all three nutrients. All fitnesses were measured relative to a common G418 sulfate‐resistant reference strain similar to the ancestral strain (Figure 1a‐d). Because of poor growth of the (nonevolved) reference strain on limiting nutrients, particularly limiting glucose, relative fitness values can be very large (e.g., Figure 1a). The reduction in nutrients had a dramatic effect on the total number of cells that can be supported in each culture (carrying capacity). Thus, nutrient limitations for the 500 generation evolution experiment were optimized such that similar numbers of cells were present in each culture. Size measurements (Beckman‐Coulter Coulter Counter Z2) indicated that glucose limitation also reduced the size of the individual cells, an observation previously noted in glucose‐limited populations of *S. cerevisiae (*Jasmin et al., 2012 *)*. Colony morphology on rich media (YPD) varied both within and between different evolved lines, with UraB^evol^ having substantially smaller colonies than any other evolved cell line and all evolved lines showing variability in colony size and morphology indicative, as expected, of heterogeneity in the evolved populations. Growth of some populations on nonoptimized media, particularly Glu^evol^ and Ura^evol^ populations on the histidine‐limited medium, generated negative fitness values (Figure 1b, d). This implies that cells were not just unable to grow during the 24‐hour competition, but that cells actually died during this time period. Trypan blue staining of Glu^evol^, His^evol^, and Ura^evol^ on limiting histidine medium shows that, indeed, the cells under conditions that garnered negative fitnesses are inviable (blue) after 24 hours, while those with positive fitnesses are viable, healthy cells after this same time period and staining procedure. Comparisons of scaled relative fitnesses across all media types (Figure 2) show that fitness is highest for each evolved population on its own evolved media (e.g., GluA^evol^ and GluB^evol^ have the highest fitness on limiting glucose) and that the GluB^evol^ population seems to have an unexpectedly high fitness on alternative nutrient limitations. As a control, fitness was also measured on YPD medium (Figure 3). Interestingly, most evolved populations have a lower fitness than the ancestral strain on rich media. Also as a control, the fitness of the ancestral strain was measured on each of the media types (Figure 1a‐d).

To analyze the rate of adaptation, we measured the relative fitness of populations evolved under single and multiple selective pressures. Each of the single pressure evolved populations (Glu^evol^, His^evol^, Ura^evol^) has higher fitness than the ancestral strain on the medium in which they evolved (Table 1). However, the populations evolved under all three pressures did not evolve a significantly increased fitness relative to the ancestral strain under any of the single pressures, with the exception of AllB^evol^, which appears to have adapted to glucose limitation (Table 1). When compared to each other, populations evolved under single selective pressures also have higher fitness than populations evolved under all three selective pressures (Table 2), with the exception of growth of the Glu^evol^ and AllB^evol^ populations on glucose‐limited media, which were not significantly different (Figure 1a). Our experimental observation is consistent with the predictions of the reduced rate adaptation under multiple selective pressures.

**Table 1:** Evolved populations have higher fitness than the ancestral strain

Limiting Nutrient	Fitness Test	*p* value	Evolved Population Fitness (mean ± *SD*)	Ancestral Strain Fitness (mean ± *SD*)
Glucose	Glu^evol^> ancestor	1.9 × 10^−7^ *	5.9 ± 4.5	0.9 ± 0.3
	AllA^evol^> ancestor	0.0057	1.9 ± 1.1	0.9 ± 0.3
	AllB^evol^> ancestor	2.8 × 10^−6^ *	4.1 ± 2.3	0.9 ± 0.3
Histidine	His^evol^> ancestor	1.3 × 10^−5^ *	2.3 ± 0.7	0.9 ± 0.1
	All^evol^> ancestor	0.97932	−0.3 ± 1.4	0.9 ± 0.1
Uracil	Ura^evol^> ancestor	1.0 × 10^−4^ *	1.8 ± 0.7	0.9 ± 0.1
	All^evol^> ancestor	0.142501	0.5 ± 1.4	0.9 ± 0.1
All‐	AllA^evol^> ancestor	7.7 × 10^−4^ *	1.8 ± 0.3	1.0 ± 0.2
	AllB^evol^> ancestor	7.7 × 10^−4^ *	6.5 ± 4.4	1.0 ± 0.2

* Significant at the Bonferroni corrected *α* < .0017238.

**Table 2:** Fitness of single pressure evolved populations is greater than multiple pressure evolved populations

Limiting Nutrient	Fitness Test	*p* value	Single Pressure Population Fitness (mean ± *SD*)	Multiple Pressure Population Fitness (mean ±* SD*)
Glucose	Glu^evol^> AllA^evol^	3.5 × 10^−4^ *	5.9 ± 4.5	1.9 ± 1.1
	Glu^evol^> AllB^evol^	0.16	5.9 ± 4.5	4.1 ± 2.3
Histidine	His^evol^> All^evol^	4.0 × 10^−7^ *	2.3 ± 0.7	−0.3 ± 1.4
Uracil	Ura^evol^> All^evol^	7.6 × 10^−4^ *	1.8 ± 0.7	0.5 ± 1.4

* Significant at the Bonferroni corrected *α* < .0017.

Next, we tested whether populations evolved on a particular limitation had higher fitness on that limitation than on other limiting nutrients (e.g., Glu^evol^ populations should have higher fitness than populations evolved on other limiting nutrients when grown on limiting glucose, Table 3). Furthermore, if there are no generalist mutations that increase fitness to multiple limiting nutrients or to batch culture conditions, then a population evolved under one limiting nutrient should have no better fitness than the ancestral strain when grown on a different limiting nutrient (Table 4). We confirmed this prediction in all cases except that populations evolved under limiting glucose evolved high fitness on limiting uracil and vice versa, suggesting that there may be generalist mutations that improve fitness with respect to both uracil and glucose limitation (Tables 3 and 4).

**Table 3:** Single pressure evolved populations have higher fitness on evolved limiting media than on other medias

Limiting Nutrient	Fitness Test	*p* value	Population Fitness Evolved on Limiting Media (mean ± *SD*)	Population Fitness Not Evolved on Limiting Media (mean ± *SD*)
Glucose	Glu^evol^> His^evol^	3.3 × 10^−8^ *	5.9 ± 4.5	0.9 ± 0.2
	Glu^evol^> Ura^evol^	0.011	5.9 ± 4.5	2.9 ± 1.1
Histidine	His^evol^> Glu^evol^	8.7 × 10^−7^ *	2.3 ± 0.7	−0.4 ± 0.8
	His^evol^> Ura^evol^	8.7 × 10^−7^ *	2.3 ± 0.7	−0.4 ± 0.9
Uracil	Ura^evol^> GluA^evol^	1.9 × 10^−4^ *	1.8 ± 0.7	1.0 ± 0.1
	Ura^evol^> GluB^evol^	0.37	1.8 ± 0.7	1.7 ± 0.5
	Ura^evol^> HisA^evol^	0.0013*	1.8 ± 0.7	1.0 ± 0.1
	Ura^evol^> HisB^evol^	3.2 × 10^−5^ *	1.8 ± 0.7	0.7 ± 0.1

* Significant at the Bonferroni corrected *α* < .0017.

**Table 4:** Most evolved populations do not have higher fitness than the ancestral strain on alternative media

Fitness Test	Limiting Nutrient	*p* value	Evolved Population Fitness (mean ± *SD*)	Ancestral Strain Fitness (mean ± *SD*)
Glu^evol^> ancestor	Histidine	1.0	−0.4 ± 0.8	0.9 ± 0.1
GluA^evol^> ancestor	Uracil	0.010	1.0 ± 0.1	0.9 ± 0.1
GluB^evol^> ancestor	Uracil	1.6 × 10^−4^ *	1.7 ± 0.5	0.9 ± 0.1
His^evol^> ancestor	Glucose	0.16	0.9 ± 0.2	0.9 ± 0.3
HisA^evol^> ancestor	Uracil	0.21	1.0 ± 0.1	0.9 ± 0.1
HisB^evol^> ancestor	Uracil	0.99	0.7 ± 0.1	0.9 ± 0.1
Ura^evol^> ancestor	Glucose	1.5 × 10^−6^ *	2.9 ± 1.1	0.9 ± 0.3
Ura^evol^> ancestor	Histidine	1.0	−0.4 ± 0.9	0.9 ± 0.1

* Significant at the Bonferroni corrected *α* < .0017.

### 3.2 Illumina high‐throughput sequencing results

**Figure 4:**
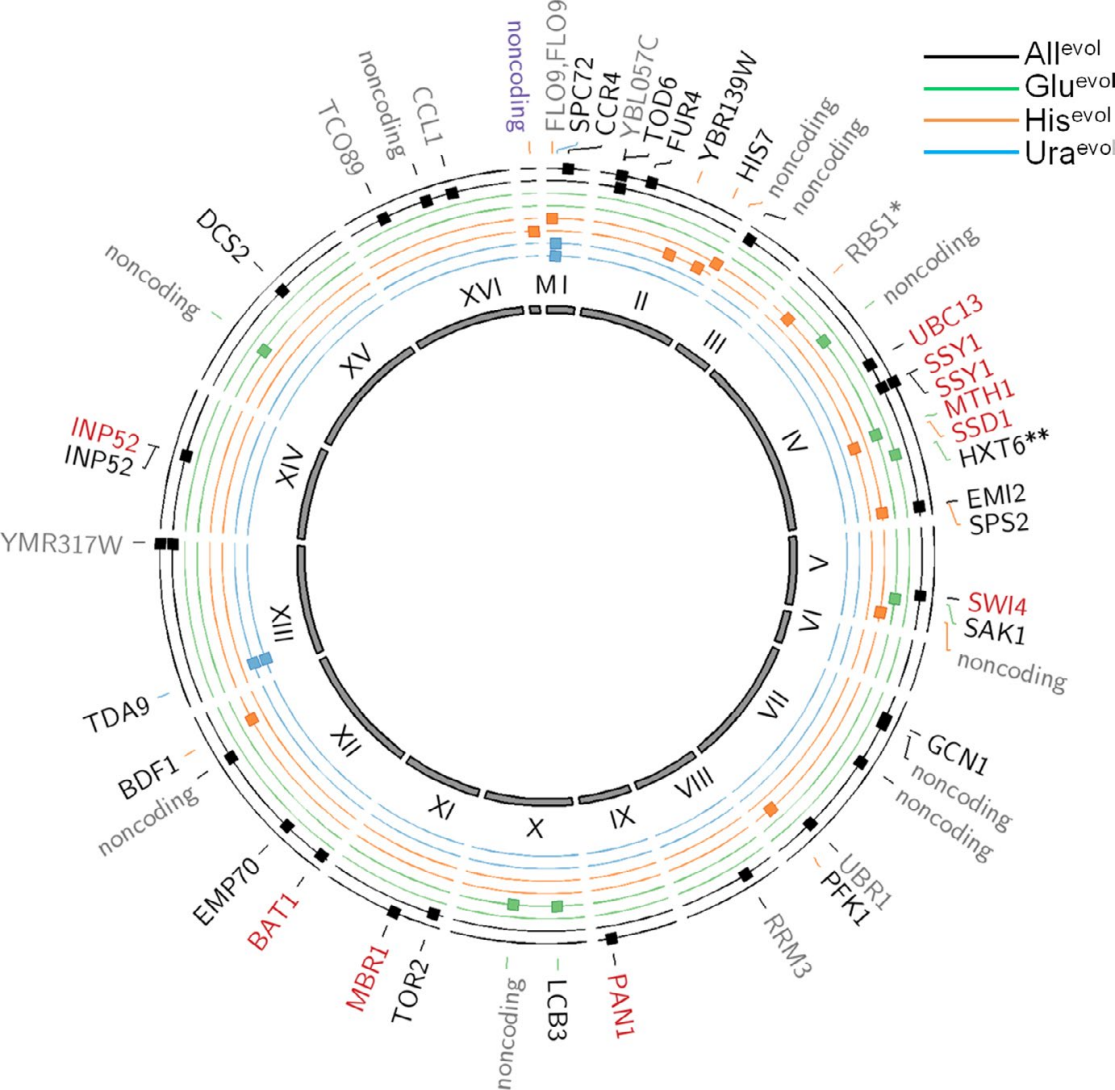
Mutations in evolved populations relative to the ancestral strain. Circles are, from exterior to interior, AllA^evol^, AllB^evol^, GluA^evol^, GluB^evol^, HisA^evol^, HisB^evol^, UraA^evol^, UraB^evol^. Inner circle (gray) displays the yeast chromosomes. Mutations: gray = mutations in noncoding regions or synonymous changes in protein‐coding genes; black = nonsynonymous mutations; red = truncation (nonsense) mutations; *=mutation in promoter region; **=amplification. Plot generated using Circos software (Krzywinski et al., 2009)

We sequenced the genomes of the eight evolved populations plus the ancestral strain. There were 675 evolved mutations across the 8 populations that were not present in the ancestral strain, 224 of which were nonsynonymous mutations in protein‐coding regions, and 67 were at >0.9 frequency in a population (Figure 4, see Tables S2‐S5 for complete list of mutations). The mutations described here are those that met stringent relevance and quality criteria. Seventeen of the 28 fixed, nonsynonymous point mutations are present in the All^evol^ populations, with many these mutations likely to be beneficial. These include mutations in genes involved in uracil transport (*FUR4*), amino acid sensing (*SSY1*), and stress response (*DCS2*), among others. Different mutations in *SSY1* (YDR160W), part of the SPS amino acid sensing system, occurred independently in AllA^evol^ and AllB^evol^. In fact, 31 genes accumulated mutations in multiple samples (Supplemental Table S5), striking evidence of convergent evolution. Eight of 17 mutations in the All^evol^ populations are nonsense mutations. We note that the strain used in our study had an intact genetic pathway for growth on glucose metabolism, but contained the deletions of HIS3 and URA3 genes, which could provide a stringent limit on the ability of these populations to grow without histidine and uracil in the medium; however, we did not see a reduction in the number of mutations on limiting histidine or uracil relative to glucose. We found only one point mutation that went to fixation in the GluA^evol^ population, though the region surrounding a glucose transport gene, *HXT6*, was amplified. Amplification of *HXT6* and another, nearly identical transporter, *HXT7* (Kruckeberg, 1996), has been reported under nutrient‐limiting conditions (Brown et al., 1998). In GluB^evol^, a negative regulator of the glucose sensing signal transduction pathway (YDR277C) was truncated via a nonsense mutation. UraA^evol^ and UraB^evol^ populations produce phenotypically distinct colony types, but have the same two underlying point mutations. This likely occurred in the growth of the original clone or in the first day's growth under uracil limitation, before replicates A and B were split. HisB^evol^ has a mutation in HIS7, an essential component of the histidine biosynthesis pathway. This is a surprise given that the gene in the histidine biosynthesis pathway, HIS3, is already deleted in the ancestral strain. However, because HIS7 is immediately upstream of HIS3, loss of function mutation in HIS7 could lower the level of 5'‐phosphoribosyl‐4‐carboxamide‐5‐aminoimidazole (AICAR), one of the His7p enzymatic products which is expected to accumulate in his3Δ populations and is known to be toxic at high levels (Rébora et al., 2005).

## 4 DISCUSSION

We have shown that we can reduce the overall rate of adaptation by simultaneously applying multiple selective pressures (Tables 1 and 2). The mechanism is consistent with the phenomenon of clonal interference, whereby beneficial mutations arise and compete within an evolving population (Gerrish & Lenski, 1998; Sniegowski & Gerrish, 2010); this phenomenon has also been examined in sexual populations (Felsenstein, 1974; Hill & Robertson, 1966; Roze & Barton, 2006). Clonal interference as originally defined requires that beneficial mutations arise in an asexual population at sufficient frequency that they compete but at a rate low enough that two interfering mutations both arise on the same ancestral background (Gerrish & Lenski, 1998); newer theory accounts for competition between clones harboring multiple beneficial mutations (Desai & Fisher, 2007; Desai et al., 2007; Sniegowski & Gerrish, 2010). Recent estimates of beneficial mutation rates in *S. cerevisiae* and cancer are sufficiently high that interference between clones is expected in evolving populations (Martincorena et al., 2018; Williams et al., 2018; Wloch et al., 2001). Intriguingly, these results may also apply to cancer and infectious diseases, though precisely where these disease systems might lie on the continuum between rare and common beneficial mutations is unknown. Our findings are likely applicable to clonal interference in cancer, however. It is a fundamental property of cancers that they harbor a multitude of mutations relative to the germ line (Hanahan & Weinberg, 2000, 2011; Salk et al., 2010). In addition, cancers are heterogeneous populations of competing clones (Maley et al., 2006; Merlo et al., 2006, 2010; Park et al., 2010), often constrained by a spatial structure such that cells only compete with their direct neighbors. Spatial structure can increase clonal interference depending on the population heterogeneity and migration rate (Campos et al., 2008; Martens & Hallatschek, 2011; Martens et al., 2011), though if there is little movement of cells, clones carrying different beneficial mutations may exist in the population but not come under direct competition. Additionally, in cancer, the local microenvironment may affect the selective effect of a particular clone (Karnoub et al., 2007). Since the doubling time of tumors is much slower than the doubling time of cancer cells *in vitro* (Friberg & Mattson, 1997), there is likely enough turnover of cells in tumors that nontoxic interventions, such as changes in nutrients and growth factors, could be used to slow the evolution of neoplastic progression.

Note that we started with a single strain that we evolved in different conditions, but have measured the fitness of the entire evolved populations, rather than cloning out strains from those populations. This provides an overall average fitness across an evolved population, but does not provide a measure of the variation in fitness among different clones within those populations. Some of that clonal variation probably generated variation in our replicates of the fitness assays (seen in Figure 1), and so was captured in our statistical tests. Tables 1 and 4 show the results of comparing these populations to the same ancestral strain.

Some results shown here may also be consistent with functional interference, a phenomenon of phenotypic interference, or trade‐off (antagonistic pleiotropy), that has also recently been linked to multi‐drug resistance (Perron et al., 2012). In our experiments, functional interference could occur if mutations conferring increased fitness in one nutrient‐limited component causes reduced fitness in another component. While we have attempted to rule out functional interference by choosing three nonoverlapping metabolic pathways, two results support this interpretation: 1) the growth of Glu^evol^ and Ura^evol^ populations, which produce negative fitnesses on limiting histidine and positive fitnesses on other environments (Figures 1 and 2) and 2) the growth of the evolved populations on rich (YPD) media, where most evolved populations show a reduced fitness compared to the ancestor (Figure 3). This suggests a trade‐off between adaptation to resource limitation and growth under abundant resources. We may also consider functional interference as a mechanism limiting the evolution of a "generalist" yeast population in the condition limiting glucose, uracil, and histidine.

We expect that the application of one vs. three selective pressures will affect the distribution of beneficial and deleterious mutations. While the total mutation rate should not change, we predicted that the three selective pressure case would create an environment in which more new mutations can have a beneficial effect, effectively skewing the distribution of the fitness effects of new mutations toward beneficial mutations and increasing the beneficial mutation supply rate. Consistent with this prediction, we see more total mutations (*p* = .04 for all mutations, *p* = .01 for nonsynonymous mutations only) that have reached fixation in the populations evolved under multiple selective pressures (Figure 4,), but consistent with clonal interference, we do not see a concomitantly increased relative fitness under three selective pressures compared to a single selective pressure (Tables 1 and 4).

It is worth noting that not all three nutrients may be limiting simultaneously over the entire course of the 500 generation evolution experiment. Rather, the fact that the AllB^evol^ population had similar fitness to the Glu^evol^ populations on glucose‐limited media suggests that, at some point during the experiment, glucose limitation was a stronger selective pressure than histidine and uracil limitations for AllB^evol^. In fact, the most limiting nutrient may cycle; as a population acquires an adaptation allowing for improved growth on one limiting nutrient, it may cease to be truly limiting until adaptations are acquired for increased fitness on the other limitations. This is similar to the R* concept from ecology (Tilman, 1982).

Regardless of whether the effect is due to clonal or functional interference, we have shown that we can slow evolution by adding selective pressures to a population (Tables 1 and 2). We may be able to take advantage of this phenomenon to design new interventions for diseases and limit the spread of resistant weeds and pests in agriculture. In fact, the addition of selective pressures is entirely consistent with the ecological approaches advocated in Integrated Pest Management (Barzman et al., 2015; Kogan, 1998). The application of the multiple selective pressures described here is a more general principle than, for example, a therapy that involves treatment with multiple bactericidal antibiotics, though this may indeed operate through a mechanism of interference (Perron et al., 2012). Selection need not be through differential cell killing, but also includes the introduction of resources that allow some clones to reproduce faster than others, perhaps through dietary interventions that change the available nutrients in the microenvironment of a tumor. In that case, all of the strains would still grow, but by altering the relative fitness of clones, particularly in the context of other limiting resources (such as space), we may be able to delay the evolution of virulence and malignancy. Because the selective pressures need not be toxic, this strategy may work well for disease prevention (e.g., cancer prevention) where subjects are relatively healthy and toxicity must be strictly limited in order to achieve a health benefit. Further investigation of this phenomenon is warranted given the potential implications for human health and agriculture.

## 5 CONCLUSIONS AND IMPLICATIONS

Human diseases, including infectious diseases and cancer, undergo adaptive evolution during disease progression. Finding a way to slow adaptation could allow us to slow the rate of disease progression and the evolution of therapeutic resistance. In this study, we tested one possible mechanism for slowing the rate of evolution in a yeast (*S. cerevisiae*) model system. Evolutionary theory predicts that if multiple mutations arise in different disease cells that are beneficial to the disease (and detrimental to the host), these mutations can compete with each other. This competition can lead to interference between these different mutations, effectively slowing the rate of adaptation. One mechanism to increase the potential for competition between beneficial mutations is to increase the number of selective pressures constraining an evolving population. In our experiment, we evolved yeast for 500 generations under either one or three selective pressures. We find that evolution under multiple selective pressures can, indeed, effectively slow the rate of adaptation.

## Supporting information

Table S1‐S5

## Data Availability

The yeast genomic sequence data that support the findings of this study are openly available in Dryad Digital Repository at https://doi.org/10.5061/dryad.0k6djh9x4
