# Supplementary material for: Application of simultaneous selective pressures slows adaptation
Source: Evol Appl. 2020 Aug 15;13(7):1615–25. doi: 10.1111/eva.13062 (PMC7484835; doi:10.1111/eva.13062)
Supplement: Supplementary file 1 — Table S1‐S5 [file EVA-13-1615-s001.docx]

**Supplemental Information**

**Supplemental Table 1: Illumina GAII Sequencing**

| **Sample** | **Mate** | **total reads** | **uniquely aligned reads** | **Unaligned reads** | **multiply aligned reads** | **Avg. Read Depth/bp** | **% bases covered** |
| --- | --- | --- | --- | --- | --- | --- | --- |
| ancestor | 1 | 7596523 | 85.99% | 2.21% | 11.80% | 23.3 | 93.35% |
| ancestor | 2 | 7596523 | 84.25% | 4.13% | 11.63% |  |  |
| AllA | 1 | 5573660 | 85.67% | 1.44% | 12.89% | 24.45 | 93.30% |
| AllA | 2 | 5573660 | 84.02% | 3.35% | 12.63% |  |  |
| AllB | 1 | 5822147 | 87.15% | 3.08% | 9.77% | 21.43 | 93.32% |
| AllB | 2 | 5822147 | 85.26% | 5.13% | 9.61% |  |  |
| GluA | 1 | 6286489 | 86.46% | 1.01% | 12.53% | 24.94 | 93.35% |
| GluA | 2 | 6286489 | 84.72% | 3.00% | 12.28% |  |  |
| GluB | 1 | 5702396 | 84.30% | 1.79% | 13.91% | 20.19 | 93.28% |
| GluB | 2 | 5702396 | 82.44% | 3.88% | 13.68% |  |  |
| HisA | 1 | 7320628 | 84.13% | 2.49% | 13.38% | 19.87 | 93.32% |
| HisA | 2 | 7320628 | 82.44% | 4.38% | 13.18% |  |  |
| HisB | 1 | 5953627 | 87.91% | 0.97% | 11.13% | 21.1 | 93.30% |
| HisB | 2 | 5953627 | 86.14% | 2.86% | 11.00% |  |  |
| UraA | 1 | 7542490 | 84.67% | 2.20% | 13.13% | 25.26 | 93.36% |
| UraA | 2 | 7542490 | 82.91% | 4.17% | 12.92% |  |  |
| UraB | 1 | 15617220 | 85.48% | 1.82% | 12.70% | 39.3 | 93.54% |
| UraB | 2 | 15617220 | 83.76% | 3.79% | 12.46% |  |  |

**Supplemental Table 2: Number of evolved SNPs / fixed SNPs**

| **Sample** | **Number SNPs** | **Number “fixed” SNPs** |
| --- | --- | --- |
| AllA | 47 | 6 |
| AllB | 111 | 31 |
| GluA | 50 | 1 |
| GluB | 59 | 7 |
| HisA | 164 | 5 |
| HisB | 55 | 3 |
| UraA | 58 | 8 |
| UraB | 131 | 6 |
| Total | 675 | 67 |

Fixed SNPs are defined as the SNPs whose frequency > 0.9.

## **Supplemental Table 3: Types of evolved SNPs**

| **sample** | **intergenic** | **noncoding** | **nonsense** | **non- synonymous** | **synonymous** |
| --- | --- | --- | --- | --- | --- |
| AllA | 17 | 0 | 4 | 18 | 8 |
| AllB | 35 | 0 | 8 | 50 | 18 |
| GluA | 20 | 0 | 2 | 18 | 10 |
| GluB | 30 | 0 | 2 | 18 | 9 |
| HisA | 73 | 0 | 4 | 60 | 27 |
| HisB | 24 | 0 | 3 | 17 | 11 |
| UraA | 28 | 1 | 1 | 18 | 10 |
| UraB | 84 | 0 | 2 | 25 | 20 |
| Total | 311 | 1 | 26 | 224 | 113 |

**Supplemental Table 4: All mutations**

| Strain | Chromo-some | Position | DNA mutation | coverage | Gene | Amino acid change | Function* |
| --- | --- | --- | --- | --- | --- | --- | --- |
| AllA^evol^ | chrI | 111712 | T>A | 21 | CCR4 (YAL021C) | D🡪V | Component of the CCR4-NOT transcriptional complex, which is involved in regulation of gene expression component of the major cytoplasmic deadenylase, which is involved in mRNA poly(A) tail shortening |
|  | chrII | 113436 | G>A | 5 | YBL057C | synonymous | One of two (see also PTH1) mitochondrially-localized peptidyl-tRNA hydrolases negatively regulates the ubiquitin-proteasome pathway via interactions with ubiquitin-like ubiquitin-associated proteins dispensable for cell growth |
|  | chrII | 281599 | G>A | 26 | FUR4 (YBR021W) | E🡪K | Uracil permease, localized to the plasma membrane expression is tightly regulated by uracil levels and environmental cues |
|  | chrIV | 776878 | G>T | 25 | SSY1 (YDR160W) | E🡪Stop | Component of the SPS plasma membrane amino acid sensor system (Ssy1p-Ptr3p-Ssy5p), which senses external amino acid concentration and transmits intracellular signals that result in regulation of expression of amino acid permease genes |
|  | chrIX | 367223 | C>A | 27 | PAN1 (YIR006C) | E🡪Stop | Part of actin cytoskeleton-regulatory complex Pan1p-Sla1p-End3p, associates with actin patches on the cell cortex promotes protein-protein interactions essential for endocytosis previously thought to be a subunit of poly(A) ribonuclease |
|  | chrXI | 264547 | C>T | 25 | MBR1 (YKL093W) | Q->Stop | Protein involved in mitochondrial functions and stress response overexpression suppresses growth defects of hap2, hap3, and hap4 mutants |
| AllA^evol^/ AllB^evol^ | chrXIII | 908216 | T>C | 5(6) | YMR317W | synonymous | Putative protein of unknown function with some similarity to sialidase from Trypanosoma YMR317W is not an essential gene |
| AllB^evol^ | chrII | 118466 | C>T | 20 | TOD6 (YBL054W) | T🡪M | Protein involved in rRNA and ribosome biogenesis binds polymerase A and C motif subunit of the RPD3L histone deacetylase complex similar to Dot6p contains chromatin specific SANT domain |
|  | chrIII | 30247 | T>A | 20 |  | noncoding | None-ARS304 (Autonomously Replicating Sequence on chromosome III**)** |
|  | chrIV | 630247 | C>T | 16 | UBC13 (YDR092W) | Q🡪Stop | Ubiquitin-conjugating enzyme involved in the error-free DNA postreplication repair pathway interacts with Mms2p to assemble ubiquitin chains at the Ub Lys-63 residue DNA damage triggers redistribution from the cytoplasm to the nucleus |
|  | chrIV | 777068 | C>A | 23 | SSY1 (YDR160W) | S🡪Stop | Component of the SPS plasma membrane amino acid sensor system (Ssy1p-Ptr3p-Ssy5p), which senses external amino acid concentration and transmits intracellular signals that result in regulation of expression of amino acid permease genes |
|  | chrIV | 1475944 | C>A | 19 | EMI2 (YDR516C) | G🡪C | Non-essential protein of unknown function required for transcriptional induction of the early meiotic-specific transcription factor IME1 required for sporulation expression is regulated by glucose-repression transcription factors Mig1/2p |
|  | chrV | 383286 | C>A | 25 | SWI4 (YER111C) | G🡪Stop | DNA binding component of the SBF complex, a transcriptional activator that regulates late G1-specific transcription of targets including cyclins and genes required for DNA synthesis and repair |
|  | chrVII | 134411 | G>T | 15 | GCN1 (YGL195W) | V🡪F | Positive regulator of the Gcn2p kinase activity, forms a complex with Gcn20p, proposed to stimulate Gcn2p activation by an uncharged tRNA |
|  | chrVII | 166742 | C>T | 11 |  | Noncoding | None |
|  | chrVII | 413970 | G>A | 15 |  | Noncoding | None |
|  | chrVII | 860281 | C>T | 18 | UBR1 (YGL195W) | Synonymous | Ubiquitin-protein ligase (E3) that interacts with Rad6p/Ubc2p to ubiquitinate substrates of the N-end rule pathway binds to proteins of the 19S particle of the 26S proteasome |
|  | chrVIII | 171743 | A>G | 22 | RRM3 (YHR031C) | Synonymous | DNA helicase involved in rDNA replication and Ty1 transposition, relieves replication fork pauses at telomeric regions |
|  | chrXI | 58415 | G>A | 18 | TOR2 (YKL203C) | A🡪V | PIK-related protein kinase and rapamycin target; subunit of TORC1, a complex that regulates growth in response to nutrients and TORC2, a complex that regulates cell-cycle dependent polarization of the actin cytoskeleton involved in meiosis |
|  | chrXII | 42043 | G>A | 30 | BAT1 (YLL048C) | Q🡪Stop | Transporter of the ATP-binding cassette (ABC) family involved in bile acid transport; similar to mammalian bile transporters |
|  | chrXII | 294730 | A>C | 20 | EMP70 (YLR083C) | L🡪V | Protein with a role in cellular adhesion and filamentous growth; 24kDa cleavage product found in endosome-enriched membrane fractions |
|  | chrXII | 789946 | G>C | 15 |  | Noncoding | None |
|  | chrXIV | 423983 | G>C | 19 | INP52 (YNL106C) | S🡪Stop | Polyphosphatidylinositol phosphatase, dephosphorylates a number of phosphatidylinositols (PIs) to PI; involved in endocytosis hyperosmotic stress causes translocation to actin patches |
|  | chrXIV | 423985 | T>A | 18 | INP52 (YNL106C) | L🡪F | Same as above |
|  | chrXV | 658289 | C>T | 23 | DCS2 (YOR173W) | P🡪L | Non-essential, stress induced regulatory protein containing a HIT (histidine triad) motif; modulates m7G-oligoribonucleotide metabolism |
|  | chrXVI | 205411 | C>T | 25 | TCO89 (YPL180W) | synonymous | Subunit of TORC1, a complex that regulates growth in response to nutrient availability; cooperates with Ssd1p in the maintenance of cellular integrity; deletion strains are hypersensitive to rapamycin |
|  | chrXVI | 464107 | G>C | 14 |  | Noncoding | None |
|  | chrXVI | 613995 | G>C | 21 | CCL1 (YPR025C) | synonymous | Cyclin associated with protein kinase Kin28p, involved in transcription initiation at RNA polymerase II promoters |
| GluA^evol^ | chrIV | 1155987-  1161421 |  |  | HXT6 | amplification | High-affinity glucose transporter of the major facilitator superfamily, nearly identical to Hxt7p, expressed at high basal levels relative to other HXTs, repression of expression by high glucose requires SNF3 |
| GluB^evol^ | chrIV | 368787 | A>G | 14 |  | Noncoding | None |
|  | chrIV | 1014801 | G>T | 19 | MTH1 (YDR277C) | S🡪Stop | Negative regulator of the glucose-sensing signal transduction pathway, required for repression of transcription by Rgt1p |
|  | chrV | 418246 | G>A | 17 | SAK1 (YER129W) | G🡪S | Upstream serine/threonine kinase for the SNF1 complex ; partially redundant with Elm1p and Tos3p; members of this family have functional orthology with LKB1, a mammalian kinase associated with Peutz-Jeghers cancer-susceptibility syndrome |
|  | chrX | 158486 | C>T | 24 | LCB3 (YJL134W) | P🡪L | Long-chain base-1-phosphate; phosphatase with specificity for dihydrosphingosine-1-phosphate, regulates ceramide and long-chain base phosphates levels, involved in incorporation of exogenous long chain bases in sphingolipids |
|  | chrX | 422267 | C>T | 6 |  | Noncoding | Ty4 long terminal repeat region |
|  | chrXV | 326692 | A>C | 25 |  | Noncoding | Centromeric region |
| HisA^evol^ | chrI | 27127 | T>C | 9 | FLO9 (YAL063C) | Synonymous | Lectin-like protein with similarity to Flo1p, thought to be expressed and involved in flocculation |
|  | chrI | 27130 | C>G | 10 | FLO9 (YAL063C) | Synonymous | Same as above |
|  | chrII | 809788 | A>T | 5 |  | Noncoding | None |
|  | chrIV | 122118 | A>G | 23 | RBS1 (YDL189W) | regulatory region | Protein of unknown function, identified as a high copy suppressor of psk1 psk2 mutations that confer temperature-sensitivity for galactose utilization; proposed to bind single-stranded nucleic acids via its R3H domain |
|  | chrIV | 1484732 | G>A | 22 | SPS2 (YDR522C) | P🡪S | Protein expressed during sporulation, redundant with Sps22p for organization of the beta-glucan layer of the spore wall |
|  | chrV | 512373 | T>C | 23 |  | Noncoding | None |
|  | chrVII | 971482 | C>G | 13 | PFK1 (YGR240C) | S🡪T | Alpha subunit of heterooctameric phosphofructokinase involved in glycolysis, indispensable for anaerobic growth, activated by fructose-2,6-bisphosphate and AMP, mutation inhibits glucose induction of cell cycle-related genes |
|  | chrXII | 921068 | C>A | 22 | BDF1 (YLR399C) | V🡪F | Protein involved in transcription initiation at TATA-containing promoters; associates with the basal transcription factor TFIID |
| HisB^evol^ | chrII | 515851 | C>A | 16 | YBR139W | T🡪N | Putative serine type carboxypeptidase with a role in phytochelatin synthesis; induced by nitrogen limitation in a GLN3, GAT1-independent manner |
|  | chrII | 715565 | A>G | 16 | HIS7 (YBR248C) | I🡪T | Imidazole glycerol phosphate synthase (glutamine amidotransferase:cyclase), catalyzes the fifth and sixth steps of histidine biosynthesis and also produces 5-aminoimidazole-4-carboxamide ribotide (AICAR), a purine precursor |
|  | chrIV | 1048428 | G>C | 19 | SSD1 (YDR293C) | S🡪Stop | Protein with a role in maintenance of cellular integrity, interacts with components of the TOR pathway; ssd1 mutant of a clinical S. cerevisiae strain displays elevated virulence |
|  | chrM | 56862 | A>C | 5 |  | Noncoding | None |
| UraA^evol^/ | chrI | 55089 | C>A | 28(41) | SPC72 (YAL047C) | K🡪N | Component of the cytoplasmic Tub4p (gamma-tubulin) complex, binds spindle pole bodies and links them to microtubules; has roles in astral microtubule formation and stabilization |
| UraB^evol^ | chrXIII | 107513 | G>A | 22(41) | TDA9 (YML081W) | G🡪S | Putative protein of unknown function; green fluorescent protein (GFP)-fusion protein localizes to the nucleus; YML081w is not an essential gene |

**All descriptions from the Saccharomyces genome database annotation (Cherry JM, Hong EL, Amundsen C, Balakrishnan R, Binkley G, Chan ET, Christie KR, Costanzo MC, Dwight SS, Engel SR, Fisk DG, Hirschman JE, Hitz BC, Karra K, Krieger CJ, Miyasato SR, Nash RS, Park J, Skrzypek MS, Simison M, Weng S, Wong ED  (2012)Saccharomyces Genome Database: the genomics resource of budding yeast. Nucleic Acids Res40(Database issue):D700-5)*

## **Supplemental Table 5: Genes with repeated mutations**

| **gene** | **gene_product** | **samples** | **times mutated** |
| --- | --- | --- | --- |
| FLO5 | flocculin FLO5 | 8 | 19 |
| FLO9 | flocculin FLO9 | 8 | 17 |
| YMR317W | hypothetical protein | 8 | 16 |
| YNL054W-B | gag-pol fusion protein | 8 | 8 |
| CAT8 | DNA-binding transcription factor CAT8 | 7 | 7 |
| FLO1 | flocculin FLO1 | 7 | 14 |
| SFP1 | zinc-coordinating transcription factor SFP1 | 7 | 7 |
| YAL064W-B/TDA8 | hypothetical protein/Tda8p | 7 | 18 |
| ATP6/tE(UUC)Q | F1F0 ATP synthase subunit a/tRNA-Glu | 6 | 14 |
| DNA2 | bifunctional ATP-dependent DNA helicase/ssDNA endodeoxyribonuclease DNA2 | 6 | 6 |
| RPL26B | ribosomal 60S subunit protein L26B | 6 | 9 |
| DOA1 | Doa1p | 5 | 5 |
| MER1/SSB2 | Mer1p/Hsp70 family ATPase SSB2 | 5 | 5 |
| PDB1 | pyruvate dehydrogenase (acetyl-transferring) subunit E1 beta | 5 | 10 |
| REG1 | protein phosphatase regulator REG1 | 5 | 10 |
| SKI3 | SKI complex subunit tetratricopeptide repeat protein SKI3 | 5 | 6 |
| UBR1 | E3 ubiquitin-protein ligase UBR1 | 5 | 7 |
| YIR018C-A/FLO11 | hypothetical protein/Flo11p | 5 | 8 |
| INO2 | Ino2p | 4 | 8 |
| MRT4 | Mrt4p | 4 | 4 |
| tA(UGC)L/YLR035C-A | tRNA-Ala/gag-pol fusion protein | 4 | 16 |
| YDR261W-B/YDR261C-D | gag-pol fusion protein/gag-pol fusion protein | 4 | 4 |
| YLL066W-B | hypothetical protein | 4 | 5 |
| DAP2/YHI9 | Dap2p/Yhi9p | 3 | 5 |
| PAU10/YRF1-1 | seripauperin PAU10/Y' element ATP-dependent helicase protein 1 copy 1 | 3 | 6 |
| PFF1/ECM8 | Pff1p/Ecm8p | 3 | 4 |
| RAD2/TNA1 | ssDNA endodeoxyribonuclease RAD2/Tna1p | 3 | 6 |
| ROX3/RPL32 | Rox3p/ribosomal 60S subunit protein L32 | 3 | 6 |
| SKY1 | serine/threonine protein kinase SKY1 | 3 | 15 |
| YIL089W/tE(CUC)I | hypothetical protein/tRNA-Glu | 3 | 4 |
| YML039W/YMD8 | gag-pol fusion protein/Ymd8p | 3 | 3 |
| YRF1-7/PAU22 | Y' element ATP-dependent helicase protein 1 copy 7/seripauperin PAU22 | 3 | 3 |

## 
